# Alien species as a potential threat for Natura 2000 habitats: a national survey

**DOI:** 10.7717/peerj.8032

**Published:** 2019-11-11

**Authors:** Joanna Perzanowska, Joanna Korzeniak, Damian Chmura

**Affiliations:** 1 Institute of Nature Conservation of Polish Academy of Sciences, Kraków, Poland; 2 Institute of Environmental Protection and Engineering, University of Bielsko-Biala, Bielsko-Biała, Poland

**Keywords:** Habitat types, Nature conservation, Poland

## Abstract

Invasion by alien species (AS) is one of the most serious threats to ecosystems. In Europe, the Natura 2000 habitats network was established to protect habitats vital for the conservation of biodiversity and function of ecosystems. Therefore, the appearance of AS in Natura 2000 habitats is a warning signal that the most valuable European habitats may be endangered. However, quantitative studies encompassing a wide spectrum of habitats are lacking, and there is no insight into the differences in the level of invasion among habitats. Our survey is based on the State Monitoring of Natura 2000 data and aimed at an assessment of the level of invasion in natural habitat types in Poland. The percentage of invaded locations, number and frequency of alien plant species was assessed in 79 Natura 2000 habitats, both terrestrial and water, investigated on 5,941 locations. The most invaded habitats (with the highest percentage of invaded plots) were dunes with *Hippophaë rhamnoides* (habitat code 2160), rivers with muddy banks (habitat code 3270), and alpine rivers and herbaceous and ligneous vegetation along their banks (habitat codes: 3220, 3230, 3240). Grassland, forest and most of the bog, mire and fen habitats and also some habitats on a rock were invaded by a relatively large number of AS, but their frequency was comparatively low. In contrast, a high frequency of AS was found in the majority of dune and costal habitats and calaminarian grasslands. Compared with the period 2000–2010, the number of AS in some riparian, grassland and forest habitats rose noticeably. The occurrence of AS showed a negative correlation with conservation status of the habitats. This study has demonstrated that standard monitoring of Natura 2000 habitats provides the basis for the detection of AS, including invasive ones, in all types of habitats, and can be used for development rapid and effective response programs.

## Introduction

The Natura 2000 European Ecological Network has been created within the territory of the European Union (EU) (International Union for Conservation of Nature , IUCN). It is a multinational conservation network designed to support the long-term survival of Europe’s most valuable species and habitats protected by EC Habitats Directive 92/43/EEC (https://ec.europa.eu/environment/nature/natura2000/index_en.htm; Evans, 2012). Each EU member country is obliged to conduct national monitoring of the conservation status of Natura 2000 habitats. The monitoring program in Poland has started in 2006. During the 13 years, 79 types of terrestrial and freshwater habitats across the entire country have been monitored.

According to Maes (2013), the conservation status of a natural habitat represents the sum of the influences on that habitat and its characteristic species, which may affect its long-term natural distribution, structure and functions, as well as the long-term survival of its characteristic species. The invasion of alien species (AS) is one important negative driver of biodiversity changes and one of the most frequently observed pressures on habitats in Europe. It increases the probability of unfavorable conservation status of natural habitats (Maes, 2013). The impact of AS on plant communities is manifested by the reduction of their species richness or proportion of typical species, i.e., a deterioration of biodiversity (Pyšek et al., 2012). Many invasive plant species transform the structure and functions of habitats by outcompeting native species for resources or changing nutrient cycling regimes (Rejmánek et al., 2005). Therefore, the national monitoring is the perfect base for mapping of the distribution of invasive species and estimating potential threats.

Currently, introductions and successful establishment of AS pose a global problem, and one of the most serious threats to ecosystems (Van Kleunen et al., 2015; Early et al., 2016; Seebens et al., 2017; Seebens et al., 2018). Combined effects of alien invasions and native extinctions may result in increasing homogenization of the floras (Winter et al., 2009). Knowledge on the distribution of AS and its spatio-temporal changes is essential for an understanding of the process of invasion. In order to control of biological invasions, building and managing large databases, such as the Global Invasive Species Database (GISD; http://www.iucngisd.org/gisd/), the Global Naturalized Alien Flora (GloNAF; https://glonaf.org/) or an information platform, such as the European Alien Species Information Network (EASIN; https://easin.jrc.ec.europa.eu/) is crucial.

However, comprehensive surveys of the occurrence of AS across the full spectrum of natural habitats over large areas are lacking (Pyšek, Chytrý & Jarošík, 2010). This is also the case for Natura 2000 sites. Such knowledge is thus essential to estimate threats and set conservation priorities for the most valuable habitats in the EU. The first in depth study in Poland summarizing the state of knowledge on alien plant species was published in 2005 (Tokarska-Guzik, 2005). Another, wider study has been published in 2012, and it was the first time when the AS were related to Natura 2000 habitats (Tokarska-Guzik et al., 2012). However, these works were not based on systematic research of Natura 2000 habitats. Due to the State Environmental Monitoring, which provides information about many components of the conservation status of Natura 2000 habitats, including the presence of AS, we can now fill this gap.

Therefore, the main aim of the study was to assess the level of plant invasion in various Natura 2000 habitats, as well as find associations between habitat types and AS occurrence. Taking into account that a barrier for alien plant invasion is biotic resistance, which is often related to habitat quality and high species diversity of plant community (Levine, Adler & Yelenik, 2004; Richardson & Pyšek, 2006; Nobis, Żmihorski & Kotowska, 2016), our specific goal was to examine whether conservation status of Natura 2000 habitats can be a predictor of biotic resistance/habitat invasibility. We hypothesized that the number, frequency and diversity of AS are negatively correlated with favorable conservation status of Natura 2000 habitats, whereas for unfavorable conservation status such correlation is positive.

## Material and Methods

This study pertains to alien plant species that arrived and established themselves in the Polish flora after the year 1500, also called neophytes, kenophytes or newcomers (Tokarska-Guzik et al., 2012). We do not deal with the oldest companions of humans, called archaeophytes or oldcomers.

Analyses are based on data from the State Environmental Monitoring for the period 2009–2018. Monitoring covered 77 terrestrial and freshwater habitats listed in Annex I of the Habitats Directive (Council Directive 92/43/EEC), and additionally two habitats being under legal protection in Poland: 91XX alder swamp forest of the *Carici elongatae-Alnetum* and 65XX eutrophic humid meadows of the alliance *Calthion*. In total, 79 types of habitats, both common and rare or endangered, were included in the monitoring (Appendix S1).

Data were collected at 5,941 locations, situated within and outside the Natura 2000 network. Observations were carried out by about 230 experts at permanent monitoring locations distributed throughout the country in the pattern reflecting the regional diversity of each habitat. As a standard, each terrestrial monitoring location was represented by a transect 200 m long ×10 m wide. Transects for aquatic habitats were smaller (50 m ×3 m). For small-area habitats (e.g., humid dune slacks, petrifying springs with tufa formation), three patches located in close proximity were studied. At each monitoring location, the conservation status of the habitat was assessed on the basis of three parameters: (1) the area of the habitat, (2) its structure and functions and, (3) conservation prospects for the habitat. The structure and functions represent these features which distinguish a given Natura 2000 habitat and determine its unique nature. The assessment of structure and functions of a habitat is composed of indices specific for this habitat. Those indices describe the most essential features of this habitat type or phenomena affecting the ecological processes of key importance for its conservation. Values of indices are evaluated in a three-level scale: favorable (FV), unfavorable inadequate (U1), unfavorable bad (U2), and provide the basis for the assessment of its structure and functions (Mróz, 2017). Due to a large number of experts participating in the monitoring program, field observations might be biased by an “observer error”. It is also possible than in natural habitats of early phenology, some of late flowering alien plant species could be omitted.

**Data analysis**

On the basis of data from the State Monitoring of Natura 2000 habitats, for each type of natural habitat the following measures of the level of invasion were determined:

– the percentage of locations with AS,
– the total number of AS (S),
– the Shannon Index (H) according to the formula (Shannon, 1948):
  \begin{eqnarray*}H=-\sum _{i=1-n}^{n}{p}_{i}{\log \nolimits }_{2}{p}_{i} \end{eqnarray*}
  where *p*_*i*_ is the ratio of the *i*th AS in the total number of studied species.
– the frequency of occurrence across all locations (F) calculated as average number of records of a AS per one location in a given habitat type.

Considering the share of invaded locations, surveyed habitats were divided into four groups: (1) weakly invaded (<20% locations invaded); (2) moderately invaded (20–39% of locations invaded); (3) strongly invaded (40–59% locations invaded); and (4) very strongly invaded (>59% locations invaded).

We examined whether there was a relationship between the level of invasion and habitat structure and functions. The level of invasion was expressed by the total number AS (S), the Shannon index (H) and frequency of occurrence of AS (F). As a measure of conservation status we adopted the percentage of locations which structure and functions were coded as favorable (SFA_FV), unfavorable inadequate (SFA_U1) and unfavorable bad (SFA_U2). According to standard methodology of State Environmental Monitoring, the presence of AS is one of the indices evaluated during the assessment of habitat structure and functions. To minimize circular reasoning, original expert values of structure and functions of Natura 2000 habitats were recalculated for all locations without taking into account the occurrence of AS.

Because the data did not meet the requirements for parametrical tests i.e., normality (Shapiro-Wilk test *p*-value >0.05), we applied the Spearman rank correlation test (Best & Roberts, 1975). The Spearman correlation coefficients were calculated and a *p*-value <0.05 was considered significant. The Holm correction was applied because of multiple tests (Holm, 1979). The statistical analyses were performed in the R software and packages *stats* and *ggplot2* (R Core Team, 2017).

## Results

**Figure 1:**
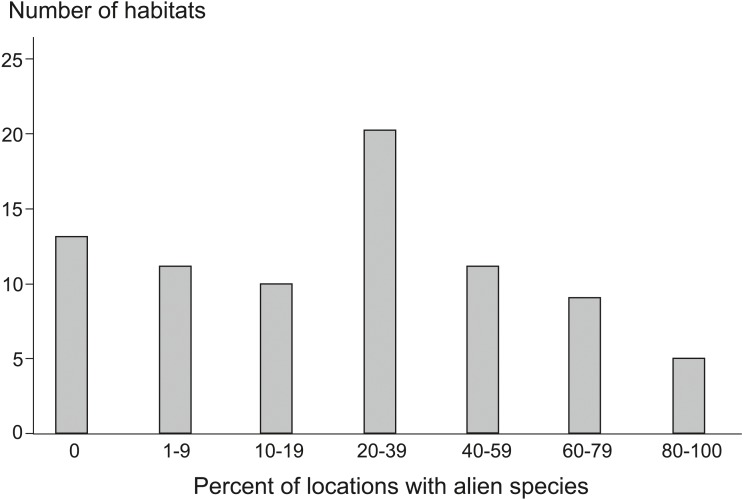
Number of Natura 2000 habitat types with different share of locations invaded by alien species. On the diagram, the habitats are grouped into classes according to the percentage of invaded locations. Habitat types within classes: 0% (without alien species): 1310, 4010, 4060, 4070, 4080, 6150, 8110, 8120, 8230, 8310, 9140, 91Q0, 9420; 1–9%: 3150, 3160, 7110, 7120, 7210, 7230, 8150, 8160, 91D0, 91T0, 9410; 10–19%: 1150, 1330, 1340, 2170, 3110, 6110, 6170, 6190, 7140, 7150; 20–39%: 2130, 2140, 2190, 3130, 3140, 40A0, 5130, 6210, 6230, 6410, 6510, 6520, 65XX, 7220, 8210, 9110, 9180, 91E0, 91P0, 91XX; 40–59%: 1210, 1230, 2110, 2180, 6430, 6440, 8220, 9130, 9170, 9190, 91I0; 60–79%: 2120, 2330, 3260, 4030, 6120, 6130, 9150, 9160, 91F0; 80–100%: 2160, 3220, 3230, 3240, 3270. For explanation of habitat codes see Appendix S1.

During monitoring (State Environmental Monitoring) of 79 Natura 2000 habitats in Poland, 117 AS were recorded at 1,879 locations (see Data S1), which means that 32% of all the surveyed locations were invaded. Alien plant species were found among 66 of the habitats, whereas in 13 we did not find any (Fig. 1). Considering the percentage of locations with AS (Fig. 1), the most strongly invaded habitats of all investigated habitat types included: dunes with *Hippophaë rhamnoides* (habitat code 2160), where AS were recorded at all monitoring plots, and rivers with muddy banks (3270), where 23 different AS were recorded 254 times (Appendix S1). Among the extremely highly invaded, i.e., habitats with more than 80% of locations invaded, there were also alpine rivers with herbaceous and ligneous vegetation along their banks (3220, 3230, 3240). From 40 to 79% of plots with AS were in some coastal habitats and dunes (1210, 1230, 2110, 2120, 2330), dry heaths (4030), xeric sand and calcareous grasslands (6120), calaminarian grasslands (6130), tall herb fringe (6430), alluvial meadows (6440) and many types of forests (9130, 9150, 9160, 9170, 9190, 91F0, 91I0). The most numerous group where were habitats with about 20 to 39% of invaded localities, included among others, some dunes (2130, 2140, 2190), grasslands and meadows (6210, 6230, 6410, 6510, 6520, 65XX), forests (9110, 9180, 91E0, 91P0, 91XX), petrifying springs (7220) and calcareous rocky slopes (8210). In many bogs, mires and fens (7110, 7120, 7140, 7150, 7210, 7230), screes (8150, 8160) and few forest habitats (91D0, 91T0, 9410) AS occurred on 1 to 19% of locations. We did not find AS in muds with *Salicornia* (1310), wet heaths (4010), alpine heaths and scrubs (4060, 4070, 4080), siliceous alpine grasslands (6150), some types of screes (8110, 8120), rocky habitats (8230), caves (8310), and some of alpine and subalpine forests (9140, 91Q0, 9420).

**Figure 2:**
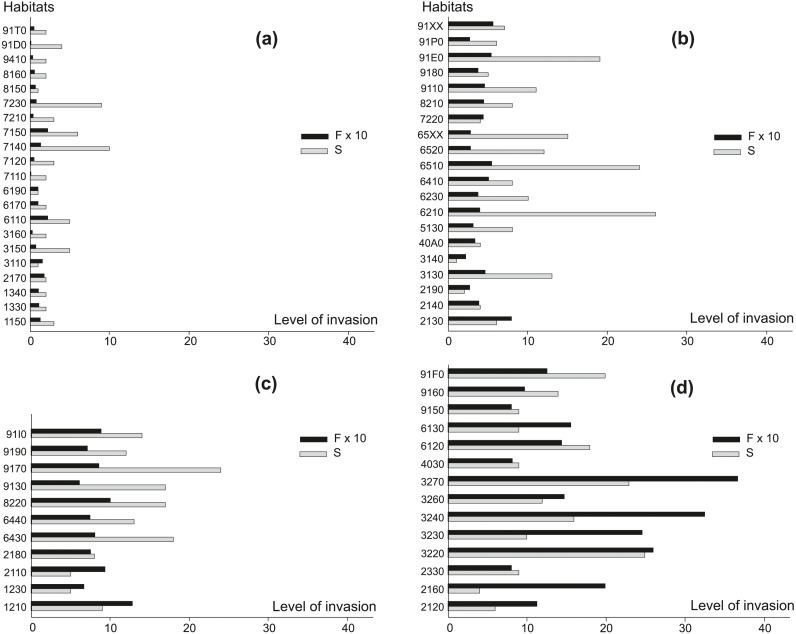
The level of invasion measured by the total number of alien species per habitat (S) and the frequency of their occurrence (F) in habitats. (A) 1–19% locations invaded, (B) 20–39% locations invaded, (C) 40–59% locations invaded, (D) 60–100% locations invaded. For ease presentation F ×10 was used. For explanation of habitat codes see Appendix 1.

Another measurement of the level of invasion was the number of all alien plant species per habitat and the frequency of occurrence of any AS in a given habitat type (Fig. 2). In habitats characterized by the smallest percentage of invaded locations (Fig. 2A), the total number of AS most often amounted to several species and their frequency was also low. Only in mires and fens (7140, 7150, 7230) between six and ten such species were recorded. Among habitats classified as moderately invaded (Fig. 2B), a high total number of AS (ten or more) was observed for: oligotrophic to mesotrophic standing waters (3130), grasslands and meadows (6210, 6230, 6510, 6520, 65XX), and few forest habitats (9110, 91E0). The highest frequency of AS (0.8) was observed in ‘grey dunes’ (2130). In the group of strongly invaded habitats (Fig. 2C), 64% of habitats were colonized by 12 or more AS, and their frequency was higher than 0.6 species per location. In this group forest habitats prevailed. A majority of AS, up to 24 species, were recorded in oak-hornbeam forests (9170). In this habitat, 0.9 AS on average were noted per location and AS occurred in more than 55% of monitoring locations (Appendix S1). In the group of habitats where AS were recorded in more than 60% of locations (Fig. 2D), the habitats associated with running waters (3220, 3230, 3240, 3260, 3270) prevailed, where AS frequency usually exceeded 1.5 per location. Rivers with muddy banks (3270) represented the most strongly invaded habitat in this group. On average, 3.7 AS were noted per location and 97% of monitoring locations in this habitat were invaded (Appendix S1). In some dune habitats (2120, 2160, 2330), European dry heaths (4030) and calaminarian grasslands (6130) only few AS were observed (4–9 taxa), but they occurred with high frequency (0.8–2.0; Appendix S1). Among very strongly invaded habitats, were also xeric sand calcareous grasslands (6120) with 76% of locations invaded by 18 AS, on average 1.4 AS per location.

In general, a very high frequency of AS compared with their total number was recorded in running waters (3230, 3240, 3270), dunes (2110, 2120, 2160) and calaminarian grasslands (6130). In contrast, grasslands and meadows (6210, 6230, 6430, 6440, 6510, 6520, 65XX), forests (9110, 9130, 9170, 91D0, 91E0, 91P0), and the majority of bogs, mires and fens (7110, 7120, 7140, 7150, 7210, 7230) were characterized by relatively high total number of AS, but occurring at relatively low frequency.

**Figure 3:**
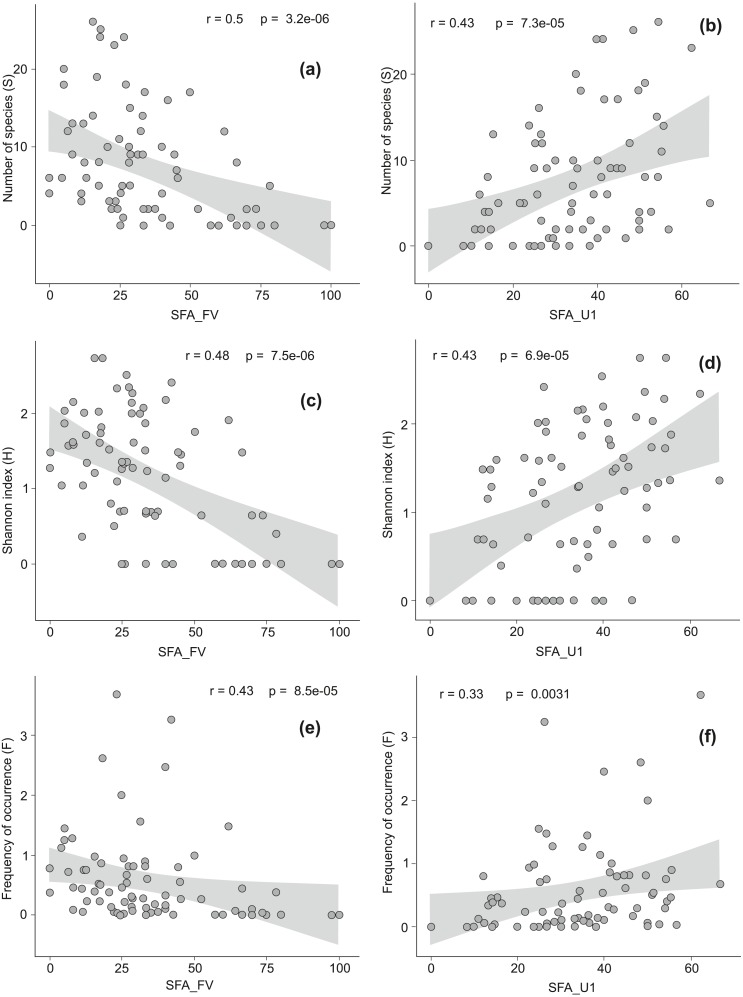
The Spearman rank correlation between the conservation status of Natura 2000 habitats and the level of invasion by alien plant species. Good conservation status is expressed by the percentage of locations with favorable structure and functions of a habitat (SFA_FV), inadequate conservation status is expressed by the percentage of locations with unfavorable inadequate structure and functions of a habitat (SFA_U1). On diagrams (A) and (B) relationship between good (A) or inadequate (B) habitat conservation status and the total number of alien species per habitat (S) is presented. Diagrams (C) and (D) show relationship between good (C) or inadequate (D) habitat conservation status and the Shannon index (H). Diagrams (E) and (F) reveal relationship between good (E) or inadequate (F) habitat conservation status and the frequency of occurrence of alien species across all locations in a given habitat type (F).

There were moderate negative correlations between favorable structure and functions of a habitat (SFA_FV) and the number of AS (S), their frequency (F) and diversity (H) (Figs. 3A, 3C, 3E). The correlation between unfavorable inadequate habitat structure and functions (SFA_U1) and S, F, and H were positive and moderate (Figs. 3B, 3D, 3F). We have not found any relationships between the presence of AS and the structure and functions in strongly distorted plots (SFA_U2). In alpine and subalpine screes (8110, 8120), alpine *Pinus cembra* forests (9420), Western Carpathian calcicolous *Pinus sylvestris* forests (91Q0) and *Tilio-Acerion* forests on slopes, screes and ravines (9180) the share of locations with favorable habitat structure and functions exceeded 75% (see Data S2). Among these habitats AS occurred only in *Tilio-Acerion* forests (9180) where 34% of locations were invaded. In case of some dunes (2110, 2120, 2130, 2140), forests (9160, 9190, 91F0), grasslands and meadows (6110, 6120, 6440), mires and fens (7150, 7230), salt marshes (1330) and annual vegetation on drift lines (1210), locations with unfavorable bad habitat structure and functions prevailed. Within this group the percentage of invaded locations was very diverse, ranging from 6 to 76% (Appendix S1).

## Discussion

The expansion of AS is a one of the major threats to biodiversity and challenges for nature conservation (e.g., Marchante, Marchante & Freitas, 2005; Botta-Dukát, 2008; Chytrý et al., 2009; Early et al., 2016; Seebens et al., 2018). Scientists have used different methods for mapping the presence of AS, but using the data from the Natura 2000 habitats monitoring program, we received detailed information at the national level. Such dataset is meaningful since Poland covers 312,696 km^2^, being the 9th largest country in Europe by area. The AS were found in 84% of the 79 habitats studied. A similar or slightly higher level of invasion was also observed in other Central European countries (Walter et al., 2005; Botta-Dukát, 2008; Pyšek et al., 2017). These results indicate the relevance of alien plant species in many habitats of the EU.

According to the theory of fluctuating resource availability (Davis, Grime & Thompson, 2000), the susceptibility of different vegetation types to invasion is positively correlated with an increase in the amount of available resources, for instance due to disturbances or periodical enrichment in nutrients (eutrophication). Our results demonstrate that in natural habitats with high resource dynamics there are more alien plant species than in these with stable levels of nutrients. The highest frequency of AS was observed for running waters: alpine rivers and the herbaceous vegetation along their banks (3220), alpine rivers and their ligneous vegetation with *Myricaria germanica* and *Salix eleagnos* (3230, 3240), as well as rivers with muddy banks (3270). The high invasibility of these habitats was confirmed in some regions in Poland (Koszela & Tokarska-Guzik, 2008; Dyderski & Jagodziński, 2016; Nobis, Żmihorski & Kotowska, 2016) as well as in other European countries (Walter et al., 2005; Vilà, Pino & Font, 2007; Botta-Dukát, 2008; Zelnik, 2012). Rivers are efficient migration routes for organisms not only because of an easy transport of propagules by water but also beacuse of natural and recurrent disturbances occurring in the habitat (Tokarska-Guzik, 2005; Richardson et al., 2007; Zając, Tokarska-Guzik & Zając, 2011; Dyderski & Jagodziński, 2016). A very important factor contributing to frequent and extensive disturbances in river valleys is also human activity causing the destruction and deterioration of aquatic habitats due to eutrophication, damming or fragmentation (Walter et al., 2005; Richardson et al., 2007). In the group of habitats with the highest percentage of invaded locations and high frequency of occurrence of AS there are some types of dunes (2120, 2160, 2330), grasslands (6120, 6130) and European dry heaths (4030). All of them belong to open habitats and are distinct because of low or very low density of vegetation, usually of pioneer character. Except of ‘white dunes’ (2120) and dunes with *Hippophaë rhamnoides* (2160) many sites of those habitats are of secondary of anthropogenic origin, e.g., calaminarian grasslands (6130) which in Poland overgrown older spoil heaps around mines. Availability of bare ground supports susceptibility of the indigenous community to invasion, especially when it coincides with disturbance and eutrophication (Burke & Grime, 1996). Relatively high number of AS within xeric sand calcareous grasslands (6120) in comparison to remaining open habitats belonging to this group, may be a consequence of considerable variability of this habitat regarding its fertility and the type and the level of disturbances. Most of the sand grasslands have formed at the area of former fields or neighbor with fields, which makes them to some extent constantly exposed to anthropogenic pressure (Botta-Dukát, 2008). At old fields, roadsides or other areas frequented by people usually some introduced species are present and they may be sources of propagule supply. Remaining types of open habitats belonging to this group are poor in nutrients which to a certain extent, restricts the number of AS connected with them. In calaminarian grasslands (6130) alien plants can be limited by toxic soil conditions considered as potential abiotic constraints on invasion (D’Antonio, Levine & Thomsen, 2001).

In alluvial forests (91E0) the level of invasion measured by the percentage of locations invaded, number of AS (S) and frequency of their occurrence (F) is lower than in other habitats associated with rivers (34%, *S* = 19, *F* = 0.5 and 80–97%, *S* = 10–25, *F* = 1.5–3.7 respectively, Appendix S1). This result can be a consequence of choosing for monitoring the most typical and thus not significantly transformed habitat patches. Nobis, Żmihorski & Kotowska (2016) suggested that forest habitats may hamper invasions in river valleys. Hence, these habitats should be monitored, because it is fairly possible that AS will increase their presence very quickly in such habitats.

A wide range of invasion levels were observed in dunes, where 18–100% of invaded locations were recorded, while the average frequency of alien species ranged from 0.2 in dunes with *Salix repens* ssp. *argentea* (*Salicion arenariae*) to 2.0 in dunes with *Hippophaë rhamnoides* (2160). Most often, i.e., in 67% of the habitats from this group, the frequency of AS was higher than 0.8. These results may be explained by the limitations of monitoring rather than the invasibility of these sites. As in many other studies (Walter et al., 2005; Vilà, Pino & Font, 2007; Botta-Dukát, 2008; Chytrý et al., 2009; Medvecká et al., 2014; Giorgis et al., 2016; Küzmič & Šilc, 2017), halophytic (developing at high salt contents in soil) and alpine habitats (on rocks, screes and in forests), caves, surface running waters, raised and blanket bogs, valley mires, poor fens and transition mires and base-rich fens proved to be the most resistant to invasion. The spread of AS in these habitat types is restricted by extreme environmental conditions (e.g., a harsh climate in high mountains, substrate salinity) and the absence of anthropogenic pressure.

The invasion level was evaluated on the basis of simple measures: the total number of AS in a given habitat (S), frequency of AS (F), and percentage of invaded locations. The first measure is the most sensitive to the emergence of new species but at the same time most variable, susceptible to transient fluctuations of species composition and ephemeral appearances of single species. Frequency of occurrence is a more robust measure. Perhaps it is more strongly linked with habitat invasibility because it is relatively higher than S in habitats with an excess of resources. The relatively high frequency compared with number of AS in open habitats (coastal, dunes, calaminarian and sand grasslands) probably results from an abundance of light and space, while along running waters (rivers with muddy banks, alpine rivers and the herbaceous and ligneous vegetation along their banks), from the high contents of nutrients and water flow related disturbances. On the other hand, in habitats characterized by strong competition for nutrients (bogs, mires, fens, rocks), light (fertile forests) or for both nutrients and light (meadows, poor forests), the total number of AS was relatively high and was accompanied by reduced frequency. The frequency of AS analyzed in combination with the percentage of invaded locations provides more accurate picture of the invasion level than S alone because of its stronger link with the sustained success of alien species.

Comparing the results obtained in the State Environmental Monitoring (2009–2018) and results published by Tokarska-Guzik et al. (2012), we found that the number of invaded types of habitat increased almost twofold (from 38 to 66). The number of AS recorded in various habitat types also rose, especially in river bank, grassland and forest habitats. Only in hydrophilous tall herb fringe communities (6430) we recorded five less AS. However, these comparisons should be interpreted with caution because of methodological differences. The study of Tokarska-Guzik et al. (2012) was mainly based on an extensive database of the Distribution Atlas of Vascular Plants in Poland (Zając & Zając, 2001) and referred to a much longer period of time. Furthermore, in contrast to the State Environmental Monitoring, natural habitats or Natura 2000 sites were not preferred in the survey. Special attention to natural habitats in the monitoring program may to a certain extent be responsible for a higher number of invaded habitat types. However an increase of the number of AS in some natural habitats is already a consequence of genuine change and proves an encroachment of AS into most valuable natural areas. These dynamic changes result both from the emergence of new alien species, and the increase in the abundance of already established AS that significantly affect habitats in which they live (Sádlo et al., 2017; Greenwood et al., 2018; Lenda et al., 2019). Such a pattern of changes is in accord with the forecasted increase in the risk of invasion of native floras due to the extension of the distribution range of different AS and the encroachment of newcomers (Lambdon et al., 2008; Tokarska-Guzik et al., 2012; Seebens et al., 2017; Seebens et al., 2018).

Our study confirmed the negative correlation between favorable habitat structure and functions and the level of invasion by AS. At the same time, no relationship between unfavorable bad habitat status and the presence of AS has been found. The structure and functions of habitat are determined by a number of interacting factors related to competition, dispersal limitation, environmental conditions, and disturbance. The surveyed natural habitats strongly differed in terms of their ecology and resistance to a wide spectrum of pressures. In the EU countries the abandonment of traditional management practices, drainage, restructuring of waterways and invasions of AS proved to be the most important drivers of decreasing probability of favorable conservation status of natural habitats (Maes, 2013). In our study alpine, undisturbed and nutrient poor habitat types were best preserved. These features are common to habitats most resistant to invasions (Davis, Grime & Thompson, 2000; D’Antonio, Levine & Thomsen, 2001). A deterioration of habitat conservation status and distortion of ecosystem structure contributes to their greater susceptibility to invasion. In this case the disturbance facilitates invasion, but under the possible opposite scenario the invading alien plants can themselves be drivers of habitat disturbance (MacDougall & Turkington, 2005).

A survey of plant invasions in nature reserves revealed that the number of AS increased with the increasing number of native species (Pys˘ek, Jarošík & Kuc˘era, 2002). The authors suggested that the native species and AS do not directly compete, and one of the most important factors explaining the alien diversity is vegetation type and location within or outside large protected areas. Gioria & Osborne (2014) pointed out that competitive advantages of AS over natives were often transient and only important at the early stages of an invasion process. Latombe et al. (2018) found that the importance of stochastic processes (such as dispersal and fluctuating environments) for structuring plant communities in protected areas was negatively correlated with residence times. According to their study, the environmental stochasticity affected species composition for AS with short residence times. Moreover, the turnover in widespread AS was surprisingly unrelated to the composition of widespread native species. These examples indicate that AS respond to environmental change similarly to the natives ones, although independently. Their presence is also the result of change in the structure and functions of habitat.

Data collected by the State Environmental Monitoring allow to identify which habitats are most strongly invaded by AS, and where active protection measures should be first implemented. Such actions should include, above all, the removal or containing the AS posing the greatest threat. The focus of protective actions within the most endangered habitats may result in an improvement of the conservation status of the natural habitats listed in Annex I of the Habitats Directive. As opposed to floristic and phytosociological databases, the State Environmental Monitoring allows for observation of the invasion dynamics owing to repeated surveys carried out at permanent locations according to a defined methodology. Apart from data on AS, it also provides an information on the conservation status of the invaded habitats. However, an efficient reduction of the spread of invasive AS requires development of a coherent strategy at larger spatial scale. The need for coordinated management of AS at European level has already been suggested by Pergl, Genovesi & Pyšek (2016). Therefore, the State Environmental Monitoring is a tool which could be effectively used for AS monitoring required by the EU Regulation (EU) No 1143/2014 (2016).

## Conclusion

The Polish State Environmental Monitoring is a valuable source of data on the AS invasion levels in Natura 2000 habitats. It provides information about the most endangered habitats and most common AS. It is useful for following the dynamics of changes in the number of habitats invaded by AS. In addition it allows linking AS frequency or richness to conservation status of Natura 2000 habitats. Knowledge on AS distribution is the starting point of actions aimed at protection and management of valuable habitats. Our survey allowed us to identify Natura 2000 habitat types in which protective measures should be first implemented.

##  Supplemental Information

10.7717/peerj.8032/supp- Appendix S1 The main measures of occurrence of alien species (AS) in Nature 2,000 habitats in Poland, according to the results of State Environmental Monitoring 2009-2018 * - priority habitat

10.7717/peerj.8032/supp-2 Data S1 The alien plant species found in Natura 2000 habitats in Poland A number of records of each species within a given habitat type is presented.

10.7717/peerj.8032/supp-3 Data S2 The assessment of structure and functions for Natura 2000 habitats in Poland The percentage of locations which habitat structure and functions were coded as favorable (SFA_FV), unfavorable inadequate (SFA_U1) and unfavorable bad (SFA_U2) is given. For some locations the habitat structure and functions haven’t been assessed (SFA_unknown).

## Additional Information and Declarations

### Data Availability

The following information was supplied regarding data availability: Raw data are available in the Supplemental Files.
