# Supplementary material for: Alien species as a potential threat for Natura 2000 habitats: a national survey
Source: PeerJ. 2019 Nov 11;7:e8032. doi: 10.7717/peerj.8032 (PMC6855207; doi:10.7717/peerj.8032)
Supplement: Appendix S1 — * - priority habitat [file peerj-07-8032-s001.doc]

Appendix 1. The main measures of occurrence of alien species (AS) in Nature 2000 habitats in Poland, according to the results of State Environmental Monitoring 2009-2018.

* - priority habitat

| **Habitat code** | **Habitat name** | **Number of monitored locations** | **Number of locations with AS** | **% of locations with AS** | **Number of AS (S)** | **Shannon index (H)** | **Total number of records of AS** | **Frequency of occurrence of AS**  **(F)** |
| --- | --- | --- | --- | --- | --- | --- | --- | --- |
| **Coastal habitats** | | | | | | | | |
| 1150 | * Coastal lagoons | 54 | 6 | 11.1 | 3 | 0.8 | 7 | 0.1 |
| 1210 | Annual vegetation of drift lines | 25 | 13 | 52.0 | 9 | 1.6 | 32 | 1.3 |
| 1230 | Vegetated sea cliffs of the Atlantic and Baltic coasts | 15 | 7 | 46.7 | 5 | 1.4 | 10 | 0.7 |
| **Halophytic habitats** | | | | | | | | |
| 1310 | *Salicornia* and other annuals colonising mud and sand | 4 | 0 | 0.0 | 0 | 0.0 | 0 | 0.0 |
| 1330 | Atlantic salt meadows (*Glauco-Puccinellietalia maritimae*) | 18 | 2 | 11.1 | 2 | 0.7 | 2 | 0.1 |
| 1340 | * Inland salt meadows | 37 | 4 | 10.8 | 2 | 0.7 | 4 | 0.1 |
| **Dunes** | | | | | | | | |
| 2110 | Embryonic shifting dunes | 31 | 18 | 58.1 | 5 | 0.7 | 29 | 0.9 |
| 2120 | Shifting dunes along the shoreline with *Ammophila arenaria* (white dunes) | 23 | 17 | 73.9 | 6 | 1.1 | 26 | 1.1 |
| 2130 | *Fixed coastal dunes with herbaceous vegetation (grey dunes) | 33 | 12 | 36.4 | 6 | 1.5 | 26 | 0.8 |
| 2140 | * Decalcified fixed dunes with *Empetrum nigrum* | 21 | 7 | 33.3 | 4 | 1.3 | 8 | 0.4 |
| 2160 | Dunes with *Hippophaë rhamnoides* | 4 | 4 | 100.0 | 4 | 1.3 | 8 | 2.0 |
| 2170 | Dunes with *Salix repens* ssp. *argentea* (*Salicion arenariea*) | 11 | 2 | 18.2 | 2 | 0.6 | 2 | 0.2 |
| 2180 | Wooded dunes of the Atlantic, Continental and Boreal region | 48 | 21 | 43.8 | 8 | 1.7 | 36 | 0.8 |
| 2190 | Humid dune slacks | 19 | 4 | 21.1 | 2 | 0.6 | 5 | 0.3 |
| 2330 | Inland dunes with open *Corynephorus* and *Agrostis* grasslands | 78 | 47 | 60.3 | 9 | 1.6 | 63 | 0.8 |
| **Standing water** | | | | | | | | |
| 3110 | Oligotrophic waters containing very few minerals of sandy plains (*Littorelletalia uniflorae*) | 45 | 7 | 15.6 | 1 | 0.0 | 7 | 0.2 |
| 3130 | Oligotrophic to mesotrophic standing waters with vegetation of the *Littorelletea uniflorae* and/or *Isoëto-Nanojuncetea* | 85 | 30 | 35.3 | 13 | 1.6 | 39 | 0.5 |
| 3140 | Hard oligo-mesotrophic waters with benthic vegetation of *Chara* spp. | 88 | 19 | 21.6 | 1 | 0.0 | 19 | 0.2 |
| 3150 | Natural eutrophic lakes with *Magnopotamion* or *Hydrocharition* - type vegetation | 287 | 16 | 5.6 | 5 | 1.3 | 21 | 0.1 |
| 3160 | Natural dystrophic lakes and ponds | 68 | 2 | 2.9 | 2 | 0.6 | 2 | 0.0 |
| **Running water** | | | | | | | | |
| 3220 | Alpine rivers and the herbaceous vegetation along their banks | 33 | 30 | 90.9 | 25 | 2.7 | 86 | 2.6 |
| 3230 | Alpine rivers and their ligneous vegetation with *Myricaria germanica* | 15 | 12 | 80.0 | 10 | 2.2 | 37 | 2.5 |
| 3240 | Alpine rivers and their ligneous vegetation with *Salix elaeagnos* | 19 | 18 | 94.7 | 16 | 2.4 | 62 | 3.3 |
| 3260 | Water courses of plain to montane levels with the *Ranunculion fluitantis* and *Callitricho-Batrachion* vegetation | 71 | 55 | 77.5 | 12 | 1.9 | 105 | 1.5 |
| 3270 | Rivers with muddy banks with *Chenopodion rubri* p.p. and *Bidention* p.p. vegetation | 69 | 67 | 97.1 | 23 | 2.3 | 254 | 3.7 |
| **Heath and scrub** | | | | | | | | |
| 4010 | Northern Atlantic wet heaths with *Erica tetralix* | 7 | 0 | 0.0 | 0 | 1.5 | 0 | 0.8 |
| 4030 | European dry heaths | 70 | 43 | 61.4 | 9 | 0.0 | 57 | 0.0 |
| 4060 | Alpine and Boreal heaths | 21 | 0 | 0.0 | 0 | 0.0 | 0 | 0.0 |
| 4070 | * Bushes with *Pinus mugo* and *Rhododendron hirsutum* (*Mugo-Rhododendretum hirsuti*) | 13 | 0 | 0.0 | 0 | 0.0 | 0 | 0.0 |
| 4080 | Sub-Arctic *Salix* spp. scrub | 10 | 0 | 0.0 | 0 | 1.2 | 0 | 0.3 |
| 40A0 | * Subcontinental peri-Pannonic scrub | 15 | 4 | 26.7 | 4 | 2.0 | 5 | 0.3 |
| 5130 | *Juniperus communis* formations on heaths or calcareous grasslands | 39 | 9 | 23.1 | 8 | 1.5 | 12 | 0.8 |
| **Grasslands** | | | | | | | | |
| 6110 | * Rupicolous calcareous or basophilic grasslands of the *Alysso-Sedion albi* | 31 | 5 | 16.1 | 5 | 1.6 | 7 | 0.2 |
| 6120 | * Xeric sand calcareous grasslands | 63 | 48 | 76.2 | 18 | 2.1 | 91 | 1.4 |
| 6130 | Calaminarian grasslands of the *Violetalia calaminariae* | 16 | 10 | 62.5 | 9 | 2.0 | 25 | 1.6 |
| 6150 | Siliceous alpine and boreal grasslands | 20 | 0 | 0.0 | 0 | 0.0 | 0 | 0.0 |
| 6170 | Alpine and subalpine calcareous grasslands | 20 | 2 | 10.0 | 2 | 0.6 | 2 | 0.1 |
| 6190 | Rupicolous pannonic grasslands (*Stipo-Festucetalia pallentis*) | 10 | 1 | 10.0 | 1 | 0.0 | 1 | 0.1 |
| 6210 | Semi-natural dry grasslands and scrubland facies on calcareous substrates(*Festuco-Brometalia*) ( * important orchid sites) | 205 | 58 | 28.3 | 26 | 2.7 | 80 | 0.4 |
| 6230 | * Species-rich *Nardus* grasslands, on siliceous substrates in mountain areas (and submountain areas, in Continental Europe) | 124 | 33 | 26.6 | 10 | 1.5 | 46 | 0.4 |
| **Meadows** | | | | | | | | |
| 6410 | *Molinia* meadows on calcareous, peaty or clayey-siltladen soils (*Molinion caeruleae*) | 125 | 43 | 34.4 | 8 | 1.7 | 63 | 0.5 |
| 6430 | Hydrophilous tall herb fringe communities of plains and of the montane to alpine levels | 125 | 53 | 42.4 | 18 | 2.4 | 101 | 0.8 |
| 6440 | Alluvial meadows of river valleys of the *Cnidion dubii* | 75 | 36 | 48.0 | 13 | 2.0 | 56 | 0.8 |
| 6510 | Lowland hay meadows (*Alopecurus pratensis, Sanguisorba officinalis*) | 339 | 130 | 38.4 | 24 | 2.5 | 184 | 0.5 |
| 6520 | Mountain hay meadows | 120 | 24 | 20.0 | 12 | 2.1 | 33 | 0.3 |
| 65XX | *Calthion* | 133 | 31 | 23.3 | 15 | 2.3 | 37 | 0.3 |
| **Bogs, mires, fens** | | | | | | | | |
| 7110 | * Active raised bogs | 165 | 2 | 1.2 | 2 | 0.7 | 2 | 0.0 |
| 7120 | Degraded raised bogs still capable of natural regeneration | 58 | 3 | 5.2 | 3 | 1.1 | 3 | 0.1 |
| 7140 | Transition mires and quaking bogs | 149 | 17 | 11.4 | 10 | 2.2 | 20 | 0.1 |
| 7150 | Depressions on peat substrates of the *Rhynchosporion* | 62 | 10 | 16.1 | 6 | 1.3 | 14 | 0.2 |
| 7210 | *Calcareous fens with *Cladium mariscus* and species of the *Caricion davallianae* | 70 | 3 | 4.3 | 3 | 1.1 | 3 | 0.0 |
| 7220 | * Petrifying springs with tufa formation (*Cratoneurion*) | 64 | 25 | 39.1 | 4 | 0.4 | 28 | 0.4 |
| 7230 | Alkaline fens | 122 | 7 | 5.7 | 9 | 2.2 | 10 | 0.1 |
| **Scree** | | | | | | | | |
| 8110 | Siliceous scree of the montane to snow levels (*Androsacetalia alpinae* and *Galeopsetalia ladani*) | 40 | 0 | 0.0 | 0 | 0.0 | 0 | 0.0 |
| 8120 | Calcareous and calcshist screes of the montane to alpine levels (*Thlaspietea rotundifolii*) | 10 | 0 | 0.0 | 0 | 0.0 | 0 | 0.0 |
| 8150 | Medio-European upland siliceous screes | 14 | 1 | 7.1 | 1 | 0.0 | 1 | 0.1 |
| 8160 | * Medio-European calcareous scree of hill and montane levels | 35 | 2 | 5.7 | 2 | 0.7 | 2 | 0.1 |
| **Rocky habitats** | | | | | | | | |
| 8210 | Calcareous rocky slopes with chasmophytic vegetation | 36 | 10 | 27.8 | 8 | 1.5 | 16 | 0.4 |
| 8220 | Siliceous rocky slopes with chasmophytic vegetation | 60 | 34 | 56.7 | 17 | 1.8 | 60 | 1.0 |
| 8230 | Siliceous rock with pioneer vegetation of the *Sedo-Scleranthion* or of the *Sedo albi-Veronicion dillenii* | 3 | 0 | 0.0 | 0 | 0.0 | 0 | 0.0 |
| 8310 | Caves not open to the public | 63 | 0 | 0.0 | 0 | 0.0 | 0 | 0.0 |
| **Forests** | | | | | | | | |
| 9110 | *Luzulo-Fagetum* beech forests | 237 | 85 | 35.9 | 11 | 1.4 | 108 | 0.5 |
| 9130 | *Asperulo-Fagetum* beech forests | 287 | 139 | 48.4 | 17 | 1.2 | 175 | 0.6 |
| 9140 | Medio-European subalpine beech woods with *Acer* and *Rumex arifolius* | 15 | 0 | 0.0 | 0 | 0.0 | 0 | 0.0 |
| 9150 | Medio-European limestone beech forests of the *Cephalanthero-Fagion* | 56 | 35 | 62.5 | 9 | 1.5 | 45 | 0.8 |
| 9160 | Sub-Atlantic and medio-European oak or oakhornbeam forests of the *Carpinion betuli* | 84 | 62 | 73.8 | 14 | 1.2 | 82 | 1.0 |
| 9170 | *Galio-Carpinetum* oak-hornbeam forests | 189 | 105 | 55.6 | 24 | 1.8 | 162 | 0.9 |
| 9180 | * *Tilio-Acerion* forests of slopes, screes and ravines | 162 | 55 | 34.0 | 5 | 0.4 | 60 | 0.4 |
| 9190 | Old acidophilous oak woods with *Quercus robur* on sandy plains | 107 | 49 | 45.8 | 12 | 1.6 | 76 | 0.7 |
| 91D0 | * Bog woodland | 254 | 4 | 1.6 | 4 | 1.3 | 4 | 0.0 |
| 91E0 | * Alluvial forests with *Alnus glutinosa* and *Fraxinus* *excelsior* (*Alno*-*Padion*, *Alnion* *incanae*, *Salicion* *albae*) | 243 | 83 | 34.2 | 19 | 2.0 | 130 | 0.5 |
| 91F0 | Riparian mixed forests of *Quercus robur, Ulmus laevis* and *Ulmus* *minor*, *Fraxinus* *excelsior* or *Fraxinus* *angustifolia*, along the great rivers (*Ulmenion* *minoris*) | 117 | 86 | 73.5 | 20 | 1.9 | 147 | 1.3 |
| 91I0 | * Euro-Siberian steppic woods with *Quercus* spp. | 87 | 45 | 51.7 | 14 | 1.9 | 77 | 0.9 |
| 91P0 | Holy Cross fir forests (*Abietetum polonicum*) | 94 | 21 | 22.3 | 6 | 1.5 | 25 | 0.3 |
| 91Q0 | Western Carpathian calcicolous *Pinus sylvestris* forests | 16 | 0 | 0.0 | 0 | 0.0 | 0 | 0.0 |
| 91T0 | Central European lichen Scots pine forests | 93 | 5 | 5.4 | 2 | 0.5 | 5 | 0.1 |
| 91XX | *Carici elongatae-Alnetum* | 102 | 40 | 39.2 | 7 | 1.3 | 57 | 0.6 |
| 9410 | Acidophilous *Picea* forests of the montane to alpine levels (*Vaccinio*-*Piceetea*) | 60 | 1 | 1.7 | 2 | 0.7 | 2 | 0.0 |
| 9420 | Alpine *Larix* *decidua* and/or *Pinus* *cembra* forests | 5 | 0 | 0.0 | 0 | 0.0 | 0 | 0.0 |
